# The effect of sleep on intrusive memories in daily life: a systematic review and meta-analysis of trauma film experiments

**DOI:** 10.1093/sleep/zsac280

**Published:** 2022-11-24

**Authors:** Per Davidson, David Marcusson-Clavertz

**Affiliations:** Department of Psychology, Lund University, Lund, Sweden; Department of Psychiatry, Massachusetts General Hospital, MA, USA; Department of Psychiatry, Harvard Medical School, MA, USA; Department of Psychology, Linnaeus University, Växjö, Sweden

**Keywords:** sleep, intrusions, memory, emotion, trauma film paradigm, PTSD, involuntary memories

## Abstract

**Study Objectives:**

To synthesize the literature on the effect of sleep versus wake on the frequency and distress of intrusive memories in everyday life after watching film clips with distressing content as a proxy for traumatic experiences.

**Methods:**

We conducted a systematic review by searching PubMed and PsychInfo. The last search was conducted on January 31, 2022. We included experimental studies comparing sleep and wake groups on intrusions using ecological diary methods, whereas studies lacking a wake control condition or relying solely on intrusion-triggering tasks or retrospective questionnaires were excluded. Meta-analyses were performed to evaluate the results. Risks of biases were assessed following the Cochrane guidelines.

**Results:**

Across 7 effect sizes from 6 independent studies, sleep (*n* = 192), as compared to wake (*n* = 175), significantly reduced the number of intrusive memories (Hedges’ *g* = −0.26, *p* = .04, 95% CI [−0.50, −0.01]), but not the distress associated with them (Hedges’ *g* = −0.14, *p* = .25, 95% CI [−0.38, 0.10]).

**Conclusions:**

Although the results suggest that sleep reduces the number of intrusions, there is a strong need for high-powered pre-registered studies to confirm this effect. Risks of biases in the reviewed work concern the selection of the reported results, measurement of the outcome, and failure to adhere to the intervention. Limitations with the current meta-analysis include the small number of studies, which comprised only English-language articles, and the fact that it was not pre-registered.

## Graphical Abstract

**Graphical Abstract:**
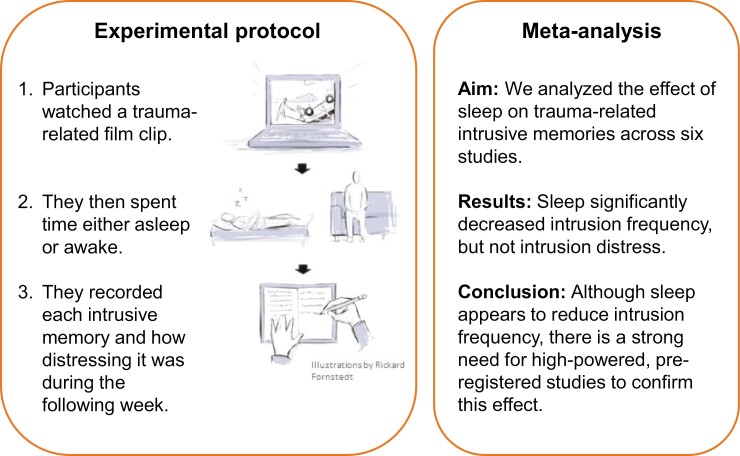

**Statement of Significance**

The current meta-analysis suggests that sleep, compared to wake, following negative emotional experiences reduces the number of intrusive memories of these experiences. Should this finding be robustly replicated in high-powered studies, this would indicate that sleep should be actively promoted in the immediate aftermath of negative emotional experiences, as this could serve a protective function that reduces their negative consequences. After extensive well-powered replication of this finding in samples with healthy participants using lab versions of traumatic experiences, the next step would be to start examining sleep-promoting interventions after real traumatic experiences. Sleep-promoting interventions are especially promising given that they are cost-effective and easy to deliver, meaning they would be applicable on a mass scale.

## Introduction

Exposure to traumatic events is often followed by involuntary, recurrent, and distressing memories of them (i.e. intrusions [1]). Both intrusions and sleep disturbances are considered hallmark symptoms of post-traumatic stress disorder (PTSD) [2].

Sleep has been found to play a beneficial role for memory consolidation [3–5]. For instance, recent meta-analytic work has revealed that sleep, as compared to wake, leads to enhanced memory performance for material encoded prior to the sleep manipulation [3, 4]. Sleep has also been suggested to moderate emotional reactivity to stressful events, although the direction of such changes has varied between studies [6]. Although research on the effect of sleep on emotional memories has typically focused on intentional retrieval of explicit memories [7–9], or emotional reactivity to reminders of the affective stimuli [6], recent research has begun examining the effect of sleep on involuntary intrusions of negative experiences [10]. The aim of this meta-analysis was to synthesize the experimental literature on the effect of sleep on intrusive memories.

Traumatic events can have long-lasting effects on people’s health and daily functioning (e.g. [11]). Not enough is yet known about the effects of sleep deprivation following distressing experiences to begin experimentally manipulating sleep after actual traumatic events or other negative experiences. Thus, work on this topic so far has been conducted using milder proxies of traumatic events, such as watching film clips depicting car crashes, bodily harm, or physical assaults, an experimental paradigm often referred to as the trauma film paradigm [12]. In this paradigm, participants first watch a trauma film and are then asked to keep a diary for a certain period of time (typically 7 days). In this diary, they are asked to record all intrusive memories of the film as soon as they experience them and to rate the degree of distress associated with each intrusion. To examine the effect of sleep on intrusions, sleep is manipulated after viewing the trauma film. This could for instance be done by having one group sleep as normal the following night and another group undergo a night of total sleep deprivation, or by having one group take a daytime nap and another group be awake for an equivalent amount of time.

The first study to examine the effect of sleep on intrusions using the trauma film paradigm found fewer intrusions during the week following a night of sleep deprivation as compared to after a night of regular sleep [10]. The authors interpreted this finding to suggest that sleep deprivation disrupts the consolidation of memories that normally occurs during sleep, leading to a reduced number of involuntary memories as well. Shortly after that paper was published, another study found an effect in the opposite direction, with fewer intrusions following sleep as compared to wake [13]. The authors interpreted this as sleep promoting the consolidation and integration of the memory, arguing that without sleep, the experience is “*predominantly laid down in memory in a disorganized and fragmented fashion that is not well integrated into its context in time, place, subsequent and previous information, and other autobiographical memories*” [13; p. 2192]. This lack of integration would then make the memory more intrusive and distressing. Since then, two more studies have also found sleep to reduce the number of intrusions [14, 15], whereas two other studies have found no group differences [16, 17] (for narrative reviews, see [6, 18, 19]). Based on the mixed findings across these studies, a meta-analysis could clarify the current status of this literature and inform future research on this topic by examining the emergent pattern of the association between sleep and intrusions with all studies combined. The previous literature has yielded two conflicting hypotheses regarding intrusion frequency. The first hypothesis states that sleep increases the number of intrusions by consolidating the memory in a manner that increases its accessibility and facilitates both voluntary and involuntary retrieval. The second hypothesis states that sleep decreases the number of intrusions by consolidating the memory in a manner that makes it less fragmented, which makes it less likely to be intrusive. By performing a meta-analysis, we sought to examine which of these hypotheses is best supported by the empirical work on this topic so far.

Beyond the number of intrusions, it is also important to consider the distress associated with them, given that all intrusive memories are not necessarily distressing [20]. The distress associated with intrusive memories is also considered a diagnosis criterion for PTSD [2], and has been suggested to be more predictive of PTSD severity than the frequency of intrusions [20, 21]. In the context of the trauma film paradigm, Kleim et al [13] found sleep to decrease the average distress of the intrusions, whereas none of the other studies has revealed any group differences [10, 14–17]. In addition to intrusion frequency, we included distress in the meta-analysis to examine if any clear pattern would emerge when aggregating all studies together. We did not have a directed hypothesis for the effect of sleep on intrusion distress.

We included studies that compared sleep to wake and measured spontaneous, involuntary memories in daily life as the outcome. The objective of this systematic review and meta-analysis was to examine if sleep, compared to wake, decreases the number of intrusions spontaneously occurring in people’s everyday life, and the average distress associated with them, in response to films with trauma-related content.

## Methods

### Literature search and inclusion criteria

The literature review for this meta-analysis followed the PRISMA guidelines (see the PRISMA 2020 Checklist in the supplemental material). The first author has previously published a narrative review on this topic [6], concluding that there were contrasting results in the literature. The present study sought to follow up on this by performing a meta-analysis and risk-of-bias assessment. The present meta-analysis was not pre-registered.

We performed a systematic search of the databases PsycInfo and PubMed, using the search terms Sleep AND Memory AND Intrusi*. The last search for both databases was conducted on January 31, 2022. To be included in the meta-analysis, a study needed to have compared a sleep and a wake group on a measure of spontaneous intrusions occurring in everyday life (i.e. outside the laboratory) recorded by the participants in some form of diary. In other words, studies examining if sleep increased intrusions during any kind of intrusion-triggering task [22, 23] were not included. Neither did we include effects related to general intrusion symptomology as measured by retrospective questionnaires [10, 15–17]. The rationale for excluding questionnaire results was that the questionnaires have been administered on different days during the week, making it difficult to compare the studies. We only included papers published in peer-reviewed journals.

**Figure 1.**
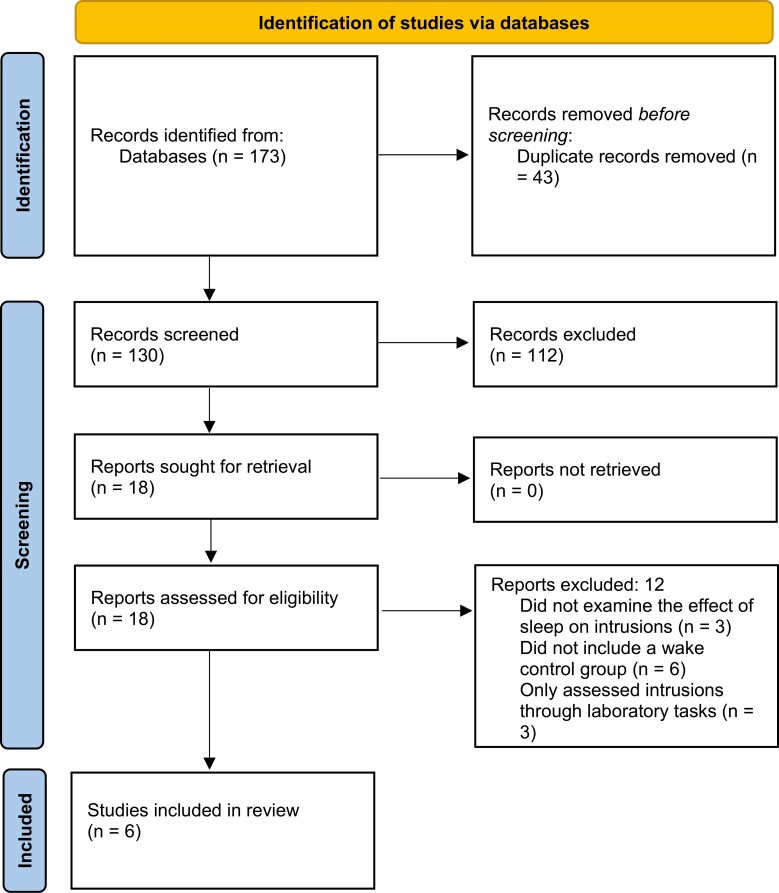
Flowchart of the literature search and exclusion of studies.

The literature search generated a total of 130 unique entries. The first author reviewed the abstract of each entry. About 112 papers were excluded during this process because they were not empirical papers or because they had not examined the effect of sleep on intrusions, leaving 18 papers for which the first author reviewed the full manuscript. Of these remaining papers, three were excluded because they had not examined the effect of sleep on intrusions, six were excluded because they did not include a wake control group, and three were excluded because they did not measure spontaneous intrusions in daily life but only included lab-based intrusion-triggering tasks, leaving 6 papers containing a total of 7 effect sizes for the final meta-analyses. For an overview of this process, see the flowchart in Figure 1.

The first author collected the relevant data (*n*, *M*, and *SD*) for each group in each study for both intrusion frequency and distress. In addition, the first author extracted the type of sleep manipulation (e.g. naps at day or regular sleep at night), sex and age of participants, and the number of participants reporting zero intrusions during the experimental week. If this information was not available, the first author contacted the corresponding author of the paper in question. We were interested in aggregated group differences across all study days, and not in day-by-day interactions (some studies have reported data for intrusion frequency on a day-by-day basis whereas others have not). Table 1 summarizes the studies included in the analyses.

**Table 1.** Overview of the studies

Study	Sleep manipulation	PSG	Trauma film content	Days^*^	Mean age	Sex	n		Number of participants with 0 intrusions		Hedge’s *g* group difference^†^	
							Sleep group	Wake group	Sleep group	Wake group	Intrusion frequency	Intrusion distress
Kleim et al., 2016 [13]	Nighttime sleep vs daytime wake or nighttime sleep deprivation (both wake groups were combined in the analysis)	Yes^‡^	“A 12-min scene from the film ‘Irreversible’. It comprised a fictional scene depicting physical and sexual violence”	6	24	F	32	33	0	0	−0.48	−0.53
Porcheret et al., 2015 [10]	Nighttime sleep vs nighttime sleep deprivation	No	“A 15 min 01 sec compilation of traumatic and distressing clips. There were six clips in total from films and television adverts, depicting scenes such as a suicide, bullying, injury, and cutting to the face”	6	22	FM	22	20	4	6	0.46	0.31
Porcheret et al., 2019 [16]	Nighttime sleep vs nighttime sleep deprivation	Yes	“A 15- min compilation of 11 traumatic and distressing clips. Scenes of a car crash, self-harm and the aftermath of genocide”	6	24	FM	24	26	7	4	−0.05	0.18
Wilhelm et al., 2021 [17]	90-minute nap opportunity (mean sleep time = 64 min) vs wake.	Yes	“A 12 min scene from the movie ‘Irreversible’… depicting a scene of explicit sexual violence”		Range: 18–35	F	33	23	NR	NR	−0.38	−0.15
Would et al., 2018 [14]												
*Dataset 1*	90-minute nap (mean sleep time = 48 min) vs wake. After viewing the movie, both groups completed a cognitive bias modification training inducing positive appraisals.	Yes	“A compilation of distressing film clips, comprising scenes displaying, for example, serious and life-threatening injuries and violence”	7	23	FM	24	22	5	4	−0.23	−0.15
*Dataset 2*	90-minute nap opportunity (mean sleep time = 35 min) vs wake. After viewing the movie, both groups completed a cognitive bias modification training inducing negative appraisals.	Yes	“A compilation of distressing film clips, comprising scenes displaying, for example, serious and life-threatening injuries and violence”	7	23	FM	27	21	4	0	−0.69	−0.57
Zeng et al., 2021 [15]	Nighttime sleep vs nighttime sleep deprivation	No	“A 14-min film... nine aversive video clips depicting fatal transportation accidents (e.g. car accidents, plane crashes, train wreck, etc)”	7	21	FM	30	30	15	10	−0.31	−0.05

PSG = Polysomnography, F = females only, FM = females and males, NR = Not Reported.

^*^ The number of days included in the analyses of group differences in intrusions.

^†^ A positive value indicates a higher mean in the sleep group. A negative value indicates a lower mean in the sleep group.

^‡^ For a subset of the participants in the sleep group only.

We included all studies that have contrasted a sleep and a wake group as long as all other factors were held equal between groups. As shown in Table 1, three studies compared nighttime sleep with nighttime sleep deprivation, one compared nighttime sleep to daytime wake or nighttime sleep deprivation (the latter two collapsed into one group for the analysis), and two were nap designs (three datasets in total). The rationale for including all these categories was to increase power by raising the sample size and that also studies with short sleep durations should be able to reveal differences between sleep and wake. We modeled the effects as random to allow differences across studies

The Woud et al [14] study administered a cognitive bias modification (CBM) training task in between the presentation of the trauma films and the sleep manipulation with one group completing a positive modification training task, and another group completing a negative modification task. We chose to include this study in the meta-analysis because even if it also included the CBM task, this was equivalent between the sleep and wake groups, and any group differences in intrusions could still be attributed to the sleep/wake manipulation. We treated the CBM positive and CBM negative conditions as two independent datasets in this meta-analysis.

### Statistical analyses

Meta-analyses were conducted in Jamovi, version 1.6.23 using the Major package version 4.0 [24]. We calculated the standardized effect size (Hedges’ *g*) by entering the mean, standard deviation, and number of participants (*n*) for each group. We used a random-effects model with restricted maximum likelihood estimator and set α to .05. Risk of bias in each study was assessed by the first author using the Cochrane criteria [25]. For this analysis, we grouped the two datasets in the Woud et al [14] study together.

We conducted sensitivity tests using the one study-removal method [26]. We further evaluated the influence of single studies with studentized residuals and Cook’s distance values. By default, Jamovi plots present scores where the Studentized residual is larger than 2 as potential outliers, but it formally considers scores above the $100\times \left(1-\frac{0.05}{2\times k}\right)\text{th}$ percentile of a standard normal distribution as outliers. Likewise, Jamovi presents any Cook’s distance score above 0.45 as potential outliers in the plot (see Supplementary Figures 1-4), but it formally considers scores above the median plus six times the interquartile range of the Cook’s distances at outliers.

Given the small number of studies, we did not examine the effect of any moderators. One study [16] reported an additional analysis in which they excluded participants in the sleep deprivation group who slept for more than 10 min, and participants in the sleep group who slept for less than 6 h. In the current meta-analysis, we included all participants from that study to make the studies comparable, and to include as many participants as possible in the analysis.

Regarding the analysis of intrusion distress, two studies [14, 15] excluded participants with zero intrusions from this analysis. Other studies [10, 16, 17] instead assigned a mean distress value of 0 to these participants. We followed the latter coding. That is, we included all participants in the distress analysis and assigned those with zero intrusions a mean distress score of 0. If statistics including all participants were not presented, the first author contacted the corresponding author of those papers to retrieve that information. One study [13] did not have any participants with zero intrusions. We were not able to obtain information about the number of participants with zero intrusions for one of the studies, but for the other studies, approximately 18% of the participants reported zero intrusions. Table 1 shows the number of participants with zero intrusions for each study. Consistent with the choices made in the original studies, we have only included intrusions occurring after the sleep manipulation. Both papers using nap designs [14, 17] started counting intrusions the day after the nap manipulation.

## Results

### Intrusion frequency

**Figure 2.**
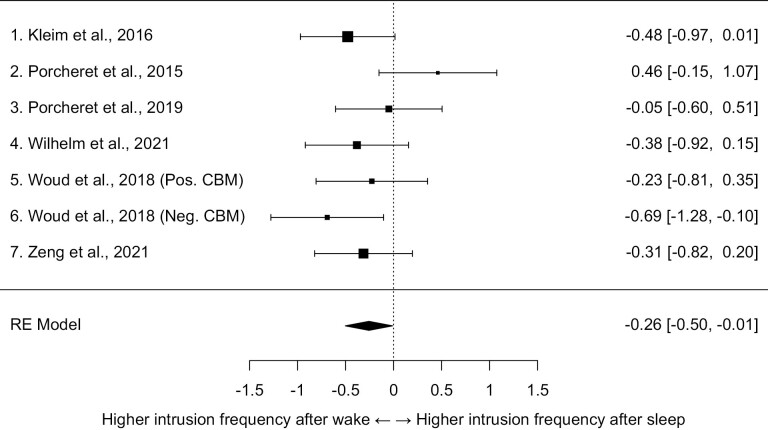
A forest plot of studies examining the standardized mean effect of sleep, as compared to wake, on the number of intrusive memories during the week after watching a film with traumatic content. Weighted mean Hedges’ *g* effect size = −0.26, *z* = −2.04, *p* = .04. Pos. CBM = Positive Cognitive Bias Modification. Neg. CBM = Negative Cognitive Bias Modification.

The random-effects model indicated a significant effect in which sleep, as compared to wake, was associated with fewer intrusions, with a small standardized mean difference (Hedges’ *g*) of −0.26, 95% CI [−0.50, −0.01], SE = 0.13, *z* = −2.04, *p* = .04. As shown in the forest plot (see Figure 2), one study had a positive estimate (more intrusions after sleep) of 0.46, whereas the others had negative estimates ranging from −0.05 to −0.69. The *I*^2^ value was 28.69%, and the *Q*-test for heterogeneity revealed *Q*(6) = 8.94, *p* = .18, indicating that the amount of heterogeneity across studies was not significant. This should, however, be interpreted with caution given the limited number of studies. Model fitting weights ranged from 11% to 18% across the studies indicating relatively equal weights. Considering the small number of studies, we did not conduct any tests for publication bias.

As a sensitivity analysis, we evaluated the influence of each study in the context of the random effects model by examining studentized residuals and Cook’s distances. We also conducted one-study removal re-analysis. The study with a positive estimate of the effect of sleep on intrusion frequency [10] was highlighted in the outlier plots as it had a studentized residual above 2 (see Supplementary Figure 1) and a Cook’s distance value above 0.45 (see Supplementary Figure 2) but it did not exceed the computed cutoffs for indicating potential outliers (which was 2.69 for the studentized residuals). Nevertheless, the effect remained significant if this study was dropped, with an estimated average standardized mean effect of −0.36 (95% CI [−0.58, −0.14], *z* = −3.17, *p* < .01). There was no significant heterogeneity for the remaining six studies, *Q*(5) = 2.89, *p* = .72, *I*^2^ = 0%, and no study was highlighted as a potential outlier. If instead of dropping this study, we dropped one of the others, the average effect estimate of the remaining six studies varied between −0.20 and −0.29, but remained significant in only one other case (if the other Porcheret et al. study [16] was dropped). The complete results of the one-study removal analysis can be found in Supplementary Data File 1. In sum, the average effect was significant with all studies included and no study exceeded the outlier cutoffs, but in 5 out of 7 cases, dropping a study yielded a non-significant estimate. Moreover, using a fixed-effects model instead of random-effects also resulted in a significant effect, Hedges’ *g* = −0.26 (95% CI [−0.47, −0.06], *z* = −2.49, *p* = .01).

### Intrusion distress

**Figure 3.**
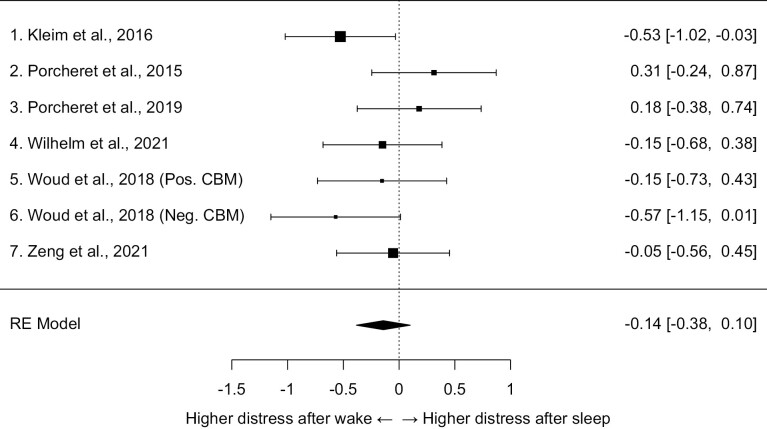
A forest plot of studies examining the standardized mean effect of sleep, as compared to wake, on the average intrusion distress during the week after watching a film with traumatic content. Weighted mean Hedges’ *g* effect size = −0.14, *z* = −1.15, *p* = .25. Pos. CBM = Positive Cognitive Bias Modification. Neg. CBM = Negative Cognitive Bias Modification.

The random-effects model for mean intrusion distress indicated no significant difference between the sleep and the wake groups. The standardized mean difference (Hedges’ *g*) was −0.14, 95% CI [−0.38, 0.10], SE = 0.12, *z* = −1.15, *p* = .25. A forest plot displaying this data is shown in Figure 3. Estimates of effect sizes ranged from −0.57 to 0.31. The *I*^2^ value was 27.99% and the *Q*-test for heterogeneity did not indicate significant heterogeneity, *Q*(6) = 8.36, *p* = .21. Again, this heterogeneity analysis should be interpreted with caution given the limited number of studies. Model fitting weights ranged from 13% to 17% across the studies indicating relatively equal weights. Considering the small number of studies, we did not conduct any tests for publication bias.

As a sensitivity analysis, we evaluated outliers and performed one-study removal re-analysis for the random effects model of distress. None of the studies exceeded the cutoffs for standardized residuals (see Supplementary Figure 3) or Cook’s distances (see Supplementary Figure 4). Dropping any single one of the studies did not change the direction or the significance of the effect with average standardized mean differences varying from −0.08 to −0.21 (all *p*-values above .066). The complete results of the one-study removal analysis can be found in Supplementary Data File 2. Using a fixed-effects model instead of random-effects resulted in a largely similar outcome (Hedges’ *g* = −0.14, 95% CI [−0.35, 0.06], *z* = −1.38, *p* = .17).

### Risk of bias analysis

The risk of bias analysis following the Cochrane criteria [25] revealed low concern for the domains regarding the randomization process, assignment to interventions, and missing outcome data. Other domains revealed higher risks. One of these was bias in measurement of the outcome. Four out of 6 studies did not include any information about if scorers of the intrusion diaries were blind to which group participants belonged to (resulting in answers of “No Information” on question 4.5 – “Is it likely that assessment of the outcome was influenced by knowledge of intervention received?”). Further, none of the studies had been pre-registered, leading to the classification of “Some concerns” for all studies regarding risk of bias in selection of the reported results.

Finally, one study [14] reported having moved participants between groups depending on if they were able to fall asleep or not, and another study [16] performed analyses with participants removed if they did not properly follow the sleep/wake instructions. This resulted in answers of “Yes” to question 2.4 on the domain related to “Risk of bias due to deviations from the intended interventions (effect of adhering to intervention)” given that the interventions were not delivered as intended (resulting in answers of “Yes” to question 2.5 as the proportion of participants who did not adhere was high enough to raise concerns, and answers of “No Information” to question 2.6 as there was no information about if this poor adherence was adjusted for in the analysis). A presentation of the risk for bias in the individual studies is presented in Table 2.

**Table 2.** Risk of biases in the individual studies according to the Cochrane criteria

	Risk of bias arising from the randomization process	Risk of bias due to deviations from the intended interventions (effect of assignment to intervention)	Risk of bias due to deviations from the intended interventions (effect of adhering to intervention)	Risk of bias due to missing outcome data	Risk of bias in measurement of the outcome	Risk of bias in selection of the reported result
Kleim et al., 2016 [13]	Low risk	Low risk	Low risk	Low risk	High risk	Some concerns
Porcheret et al., 2015 [10]	Low risk	Low risk	Low risk	Low risk	High risk	Some concerns
Porcheret et al., 2019 [16]	Low risk	Low risk	High risk	Low risk	Low risk	Some concerns
Wilhelm et al., 2021 [17]	Low risk	Low risk	Low risk	Low risk	High risk	Some concerns
Woud et al., 2018 [14]	Low risk	Low risk	High risk	Low risk	High risk	Some concerns
Zeng et al., 2021 [15]	Low risk	Low risk	Low risk	Low risk	Low risk	Some concerns

## Discussion

The current meta-analysis investigated the effect of sleep on intrusive memories in daily life by examining experimental studies using the trauma film paradigm with intrusion diaries as outcomes. The results revealed that sleep was associated with fewer intrusions, but did not reveal a significant association between sleep and the average distress of those intrusions. The results are consistent with the view that sleep reduces the number of intrusions by promoting the consolidation and integration of memories [13]. It is important to acknowledge that the effect of sleep on intrusion frequency was small, and based on a low number of studies, making it difficult to evaluate heterogeneity and publication biases. However, if this effect can be reproduced in pre-registered high-powered studies, sleep is a promising target for trauma-related interventions seeking to reduce the number of intrusions.

### Sleep and intrusion frequency

Insofar as sleep reduces intrusion frequency, an important task for future research is to identify what mediates this effect. Two putative explanations (that are not mutually exclusive) are that the effect is driven by sleep consolidating trauma-related memories, or by sleep supporting cognitive control processes (e.g. the ability to regulate which memories reach awareness). The cognitive control account would imply that an increased number of intrusions after sleep deprivation stems from a generalized deficiency in controlling which thoughts or memories are consciously retrieved. Consistent with this, some studies have observed that poor sleep is associated with a general increase in spontaneous, unguided thoughts, including mind wandering [27–31]. For instance, a daily diary study [30] found increased mind wandering following nights of self-reported poor sleep, whereas an experimental study [31] found increased mind wandering during a task with high perceptual load after a night of sleep deprivation compared to a night of regular sleep. Dietch et al [28] found an increase of intrusive memories in people suffering from PTSD on days after nights of poor sleep several years after their traumatic experience. These findings suggest that restricted sleep increases our tendencies to engage in spontaneous, task-unrelated, and intrusive thoughts, unrelated to the consolidation of a particular memory. Furthermore, Harrington et al [32] found that a night of sleep deprivation impaired the ability to voluntarily suppress the retrieval of a memory when exposed to a reminder of it. As the encoding in this study occurred the night before the sleep manipulation, it is, however, difficult to determine if this effect was caused by sleep deprivation affecting suppression ability, or by poor consolidation of the memory due to sleep deprivation. A cognitive control account would argue for sleep being important for mental health and for avoiding intrusive thoughts in general, but that it is less time-sensitive and does not necessarily need to be promoted in the immediate aftermath of a negative experience. Beyond cognitive control ability, it is important to consider that sleep deprivation causes an increase in negative mood [33], which might potentially also drive an increase in intrusions.

One finding that is arguably more consistent with the consolidation account, is the one indicating that a 90-minute nap reduced the number of intrusions [14]. The lack of a nap in the control group is unlikely to have caused any cognitive control impairments or robust changes in mood during the following week that would increase the number of intrusions. Similarly, one of the groups in the Kleim et al [13] study was not deprived of sleep over night but instead just spent a day awake after viewing the trauma film, which did not result in more intrusions than in the group that spent a night awake (but still in more intrusions than in the group who slept overnight after viewing the film). Such effects are rather consistent with an account in which post-encoding sleep decreases the number of intrusions through memory consolidation processes. There are currently too few studies to examine the type and length of sleep as moderators. As more studies are being conducted, however, it will be informative to explore to which extent such factors influence the effect.

Another way to examine the effect of sleep on intrusions not related to the consolidation of a particular memory is to examine during which days of the week following the sleep manipulation the effect is stronger. So far, no robust pattern has emerged indicating that the effect would be larger at the beginning or at the end of the experimental week. The only study finding more intrusions after sleep [10] showed that this increase was only present during the first two days. Among the studies finding fewer intrusions after sleep, Kleim et al [13] found significant decreases during day 3, 6, and 7, whereas Zeng et al [15] found significant decreases during day 1–4 (day 1 being the day after the sleep manipulation). Wilhelm et al [17] found no interaction between day and group. Would et al [14] did not report whether the effects varied depending on day, and Porcheret et al [16] only did so in the analyses in which they excluded outliers. In the future, it will be informative to examine to what extent the effects remain during the second half of the week when the acute effects of sleep deprivation on cognitive control ability and mood should have dissipated. We encourage authors to report descriptive statistics of the number of intrusions for each day separately to facilitate meta-analytic evaluations of which days during the experimental week that any group differences are present. One issue concerning the duration of sleep effects is that the number of intrusions typically decreases as a function of time, suggesting that there might be floor effects making it difficult to detect group differences during the end of the week.

To address the issue of how time-sensitive the effect of sleep is, we have a few suggestions for future research. One is to add daily or momentary measures of cognitive control ability, general tendencies to mind-wander (i.e. also of the kind not related to the trauma film), and mood throughout the experimental week. Doing so would allow for the examination of to what extent the effects are driven by the influence of sleep on these factors, by testing if any effects are still present after controlling for group differences on these control variables. Recent research has shown that brief ambulatory measures administered through smart phone devices can be used to reliably assess within-person changes and between-persons differences in cognitive abilities [34].

### Sleep and intrusion distress

Sleep did not significantly affect the mean distress associated with intrusive memories in the current meta-analysis. This is perhaps not so surprising considering that studies on the effect of sleep on emotional reactivity to reminders of emotional experiences have revealed contradictory results [6]. For this meta-analysis, we assigned a mean intrusion distress value of zero to participants who did not have any intrusions at all during the experimental week. Although it seems reasonable to us to assume that participants with zero intrusions experienced minimal intrusion-related distress, this approach may confound frequency and distress. As we found fewer intrusions in the sleep groups, there would be more participants with an imputed value of zero in these groups, making it more likely to find lower intrusion distress after sleep. We did not, however, find such an effect even when using this method, and it is feasible that the effect would be even smaller if excluding participants with zero intrusions. Considering that there were more intrusions in the wake groups, however, it should be noted that the cumulative amount of intrusion distress experienced during the experimental week was higher after wake, even if the average intrusion was not more distressing.

Future studies could extend this research by adding physiological indicators of intrusion distress, such as heart rate and skin conductance levels, which would rule out self-report biases affecting the results. This could be especially fruitful in combination with wearable devices that enable participants to report experiences of intrusive memories at the moment they occur (i.e. ambulatory assessments). Physiological arousal can then be event-locked to these intrusive memories to examine if sleep loss increases arousal in general or specifically in association with intrusive memories. Continuous physiological measures could also inform us on the temporal patterns of intrusions (e.g. the onset and duration of intrusion-related distress).

### Risk of bias

There are a few concerns regarding the risk of bias that merit discussion. The first concern relates to the risk of bias in selection of the reported result. A major issue is that none of the studies have pre-registered their analysis. Several studies also used multiple measures of similar outcomes, such as the use of impact of events scales in addition to the diary reports [10, 15–17], which increases the risk of false discoveries if *p*-values are not adjusted for multiple comparisons. A comparison of effect size estimates from traditional meta-analyses and pre-registered multi-laboratory collaborations across 15 psychological fields indicated that the former yielded almost three times larger effect sizes than the latter [35]. It is therefore critical to address this concern in future research. The second concern relates to the risk of bias in the measurement of the outcome, as four out of six studies did not include any information about the scorers of the intrusion diaries being blind to which groups participants belonged to.

The third concern relates to the risk of bias due to deviations from the intended interventions (effect of adhering to intervention). In one of the studies [14], coders changed the group assignment of participants depending on whether they fell asleep or not (e.g. assigning “wake” to those participants from the “sleep” group who were not able to fall asleep and analyzing these participants as if they were always part of the “wake” group). Generally, this re-assignment of cases across groups is a threat to internal validity as the groups can no longer be considered equivalent pre-intervention (i.e. their assignment to conditions is no longer purely the result of the randomization procedure). Another study removed several participants from one of their analyses because the participants did not properly follow the sleep instructions [16]. This attrition could cause a bias where not being able to fall asleep could be a sign of having been negatively affected by the film, and such a procedure would thus risk decreasing the number of intrusions in the sleep group. It is unfortunate that the other four studies have not reported any statistics on this, especially as the Porcheret et al [16] study that analyzed the results both with and without the non-adherers showed indications that there were significant differences between them. Masking the intervention of sleep to the participants themselves is of course impossible (as there is no such thing as placebo sleep), but we did not consider it likely that awareness of being in the sleep or wake group would have resulted in any deviations from adhering to the intended intervention following the Cochrane criteria [25].

### The need for high-powered studies

One major challenge for future research on this topic is to obtain adequate power to detect sleep effects on intrusions. The mean effect size of sleep on intrusion frequency was −0.26, i.e. a small effect size. Detecting such an effect in a between-groups design with 80% power and an alpha level of .05 using a one-tailed *t*-test would require a sample size of 184 participants per group. Detecting a similar effect size using a within-subject design, assuming a within-person correlation of *r* = .50, would require 93 participants in total for 80% power (one-tailed). Even if the study with a positive estimate [10] was excluded, a mean effect size of −0.36 would require 97 participants per group in a between-groups design, and 50 participants in total in a within-subjects design, to obtain 80% power (one-tailed). With the largest study so far including 65 participants in total, previous work on this topic may have been underpowered. This prevalence of underpowered studies is consistent with other meta-analytic studies on sleep and memory [3, 4].

Multi-laboratory collaborations may be the best approach to obtain adequate sample sizes in the future and should be a high priority for research groups working on this topic. Well-powered studies would be able to determine a much more precise estimate of the actual effect size, with a small confidence interval, which could inform us whether the effect is large and reliable enough to start recommending sleep interventions after actual potentially traumatic experiences. Such well-powered studies would also provide opportunity to robustly examine if participant characteristics such as age or sex moderate the results. It is also important to address the concerns raised in the risk-of-bias-assessment to increase our confidence in the internal validity of the findings.

There are also several other methodological differences that would be interesting to examine as potential moderators of the effect of sleep on intrusions. These include whether the sleep and sleep deprivation interventions occurred in the lab or at home, if polysomnography was implemented, the content of the trauma films, the method used to determine if an intrusion stemmed from the trauma film, and how narrowly an intrusion was defined. Considering the low number of studies, and that we are not aware of any theoretical accounts of how these factors could moderate the effects, we did not assess these moderators in the present meta-analysis. Another factor that could influence the effects is whether studies included additional interventions designed to decrease intrusion frequency such as the CBM training in the Would et al. study [14]. The CBM training in that particular study, however, did not affect intrusions as evident by there being no main effect of CBM or interaction between CBM and sleep. Another possibility is that there is substantial heterogeneity of effect sizes across these studies that we were unable to detect in this meta-analysis, but then a clear rationale would be needed on why a larger effect size than 0.26 could be expected, such as by proposing a theoretically justified moderator.

### Other work on sleep and intrusive memories

In the current meta-analysis, we only included studies that compared a sleep and a wake group and measured spontaneous, involuntary memories in daily life with diary reports. The rationale for this is that we wanted the studies to be as comparable to each other as possible, and that there were too few studies to meaningfully examine various moderators. A few studies that used other instruments and designs to address similar research questions, however, should also be acknowledged. Three studies have measured intrusions through lab-based tasks [15, 22, 23]. Consistent with the results of our meta-analysis, one study found that normal sleep, as compared to partial sleep deprivation, decreased the number of intrusions during an intrusion-triggering task in which auditory fragments of the traumatic picture story encoded the night before were being replayed the next morning [22]. However, another study comparing daytime wake to nighttime sleep using the same task did not reveal any group differences [23]. In addition to the diary results evaluated in the present study, Zeng et al [15] found fewer intrusions in the sleep group compared to the sleep deprivation group in a task that was performed the morning after the post-encoding night in which participants were asked to close their eyes for five minutes and press the space bar every time they experienced an intrusion.

Little is known about what mediates the association between sleep disturbances and intrusive memories and general PTSD development. As mentioned above, some candidate explanations are the role of sleep in memory consolidation and emotional regulation, where poor sleep would impair these processes and thus result in more intrusions and a general decrease in the ability to cope with the traumatic experience [18, 28, 36]. For non-experimental work, it is also important to consider causality in the other direction, where the hyperarousal and increased degree of nightmares and intrusive and ruminative thoughts might impair sleep quality [36, 37]. See also [38] for a study not finding any correlation between sleep difficulties two weeks prior to viewing the trauma film and subsequent intrusion frequency.

Moreover, the current meta-analysis did not examine the effect of particular sleep stages, total sleep time, or sleep quality on intrusive memories. Several studies have examined the effects of particular sleep stages on intrusion frequency and distress, but no clear patterns have emerged (see [6] for a summary of these findings).

### Implications and clinical perspectives

In summary, the current meta-analysis found sleep to reduce the number of intrusive memories of trauma films during the following week. Should this effect be replicated in more studies with larger sample sizes, it would support the beneficial role of sleep in the immediate aftermath of negative emotional experiences, such as traumatic events, as sleep could make the memory of them less intrusive.

Intrusions are a core feature of PTSD symptomology [2]. Some work has shown that re-experiencing symptomology after a traumatic experience predicts PTSD severity later on [39, 40]. Other studies have, however, found limited predictive value of early intrusion symptoms on later PTSD severity [41, 42]. An important task for future research is to further examine if such an association exists, and if it does, to determine if there is a causal connection between early intrusions and later PTSD, or if early intrusions simply serve as a marker of having been more negatively affected by the event. Even if early intrusions close to a traumatic event should turn out not to be causally related to later PTSD, it is nevertheless still important to reduce them, as they can cause a lot of distress and make it difficult to focus on everyday tasks such as school or work activities. This becomes especially important considering that sleep quality is often impaired in hospitals and intensive care units (for a review, see [43]), where many of those experiencing the most severe traumatic events are likely to end up.

It is important to develop immediate interventions that can help us cope with traumatic experiences and other negative life events in the most adaptive manner possible (see [44] for a review on memory modification interventions following traumatic experiences). There are successful treatments available for patients who have developed PTSD, but it would be particularly helpful to develop interventions that prevent these symptoms from appearing in the first place [45]. It would also be helpful to develop an intervention that is cost-effective and easy to deliver, so it can be implemented on a mass scale [46]. The result of the current meta-analysis shows that actively promoting sleep close to the traumatic event has the potential to serve as such an intervention.

Another important task for future research is to examine if sleep quality and quantity can be improved to yield an even stronger reduction in intrusion symptomology. Such proposed sleep-improving interventions include relaxation exercises, sleep hygiene education, cognitive behavioral therapy for insomnia, pharmacological therapies, and transcranial direct current stimulation [36]. Another potential intervention could be to manipulate the memories by re-activating them during sleep through targeted memory re-activation. See van der Heijden et al [47] for a review and discussion of the potential of this, as well as some potential conceptual and methodological challenges.

### Limitations

A few limitations of this meta-analysis need to be acknowledged. The review itself was not pre-registered, and the first author was already familiar with the results of the included studies [6]. A prospective meta-analysis that pre-registered the time frame and the analytic choices would thus be an important contribution to mitigate concerns of risks of biases. Another limitation is that the included studies are all part of the English literature. This has also been the case in previous narrative reviews [6, 18, 19], but it is possible that relevant non-English studies have been omitted. Further, the low number of studies to this date prevents meaningful evaluations of publication biases in this field. These limitations notwithstanding, we maintain that our review can contribute to future studies by providing information for statistical power analyses (preferably for pre-registered multi-lab collaborations) and by highlighting some risks of biases.

Regarding the issue of external validity, it is unclear how well the findings from the trauma film paradigm generalize to responses to actual traumatic experiences in clinical populations. Viewing a film clip does not entail any physical threat to the viewer and is unlikely to result in negative assumptions about oneself or the world. Caution should also be taken against generalizing the effect of sleep on intrusion frequency to the role of sleep in the development of PTSD. Michael et al [21] showed that factors other than frequency and distress of intrusions in the early aftermath of traumatic events were more predictive of the development of PTSD. These included the degree of “nowness” (i.e. re-experiencing the event as if it was happening again) and lack of memory for the context surrounding the memory (i.e. experiencing the memory as disjointed from what happened before and after it). As the trauma film clips likely induce lower degrees of nowness, it is unclear how well they generalize to general PTSD symptomology. It would be informative to include measures of nowness and contextual features of the intrusive memories in future studies. It should also be noted that the 5th edition of the DSM manual [2] considers repeated exposure to negative emotional content through various forms of media in one’s line of work as potential traumatic stressors. Studies using the trauma film paradigm should have especially high ecological validity for that kind of trauma exposure (for a discussion of this issue, see [12]). Intrusions recorded with the diary method correlate well with intrusion symptomology as measured by validated clinical scales also in clinical PTSD groups [48].

Another outstanding question regarding external validity is how well the sleep manipulations used in the present studies generalize to sleep problems in PTSD. It has been found that both too much and too little sleep the night after an actual potentially traumatizing experience is associated with increased intrusion frequency, suggesting that sleep can be relevant for intrusion symptomology also after real-world experiences [39].

## Supplementary Material

zsac280_suppl_Supplementary_Material

zsac280_suppl_Supplementary_Data

zsac280_suppl_Supplementary_File_S1

zsac280_suppl_Supplementary_File_S2

## Data Availability

All the data and code used to conduct these meta-analyses (*n*, mean and *SD* for each group in each study), and the complete one study-removal method results can be found in the supplementary data files for Jamovi.

## Preprint Repositories

A previous version of this manuscript has been uploaded at https://psyarxiv.com/dwrhe.
