# Supplementary material for: The effect of sleep on intrusive memories in daily life: a systematic review and meta-analysis of trauma film experiments
Source: Sleep. 2022 Nov 24;46(2):zsac280. doi: 10.1093/sleep/zsac280 (PMC9905779; doi:10.1093/sleep/zsac280)
Supplement: zsac280_suppl_Supplementary_Material [file zsac280_suppl_supplementary_material.docx]

**Title: The Effect of Sleep on Intrusive Memories in Daily Life: A Systematic Review and Meta-analysis of Trauma Film Experiments**

**Authors:** Per Davidson^a,b,c^ & David Marcusson-Clavertz^d^

^a)^ Department of Psychology, Lund University, Sweden.

^b)^ Department of Psychiatry, Massachusetts General Hospital, MA, USA.

^c)^ Department of Psychiatry, Harvard Medical School, MA, USA.

^d)^ Department of Psychology, Linnaeus University, Sweden.

**Corresponding author:**

Per Davidson

Department of Psychology, Lund University

Box 213

221 00 Lund

Sweden

E-mail: Per.Davidson@psy.lu.se

**Supplementary Information**


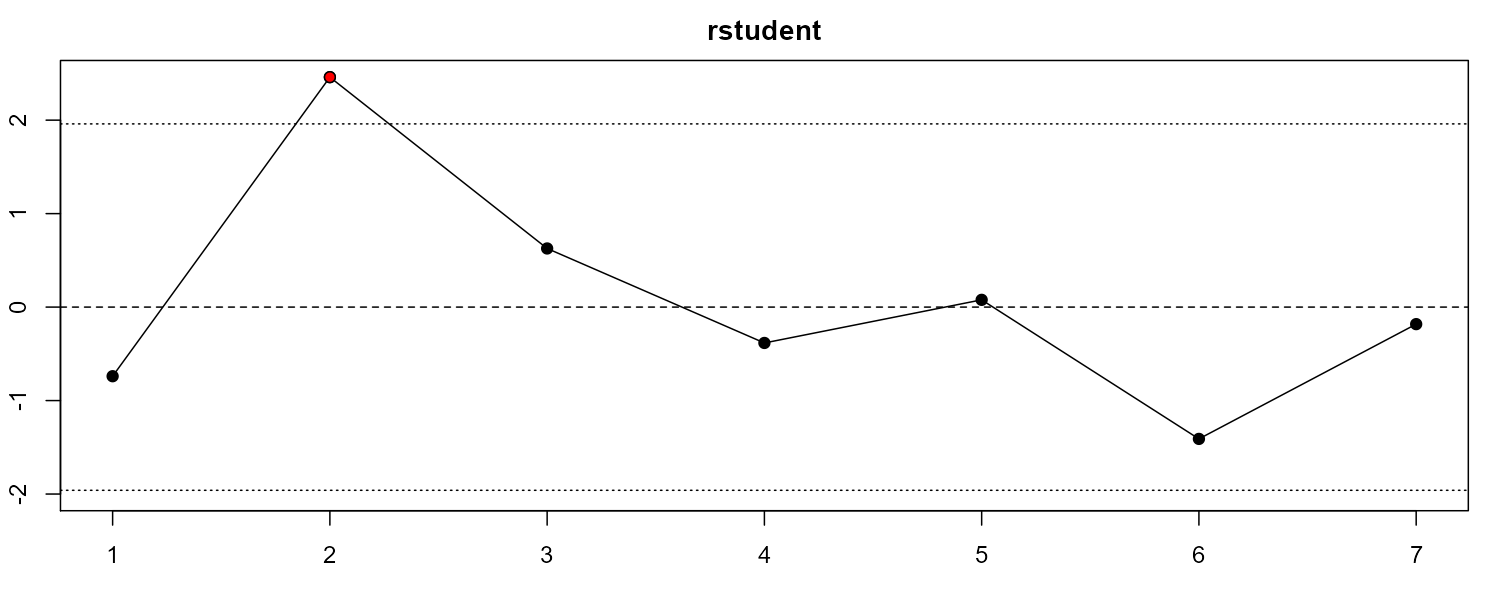


**Supplementary Figure 1.** *Standardized residuals based on the random effects model of intrusion frequency. 1. Kleim et al., 2016 [1] 2. Porcheret et al., 2015 [2] 3. Porcheret et al., 2019 [3] 4. Wilhelm et al., 2021 [4] 5. Woud et al., 2018 Positive Cognitive Bias Modification [5] 6. Woud et al., 2018 Negative Cognitive Bias Modification [5] 7. Zeng et al., 2021 [6].*


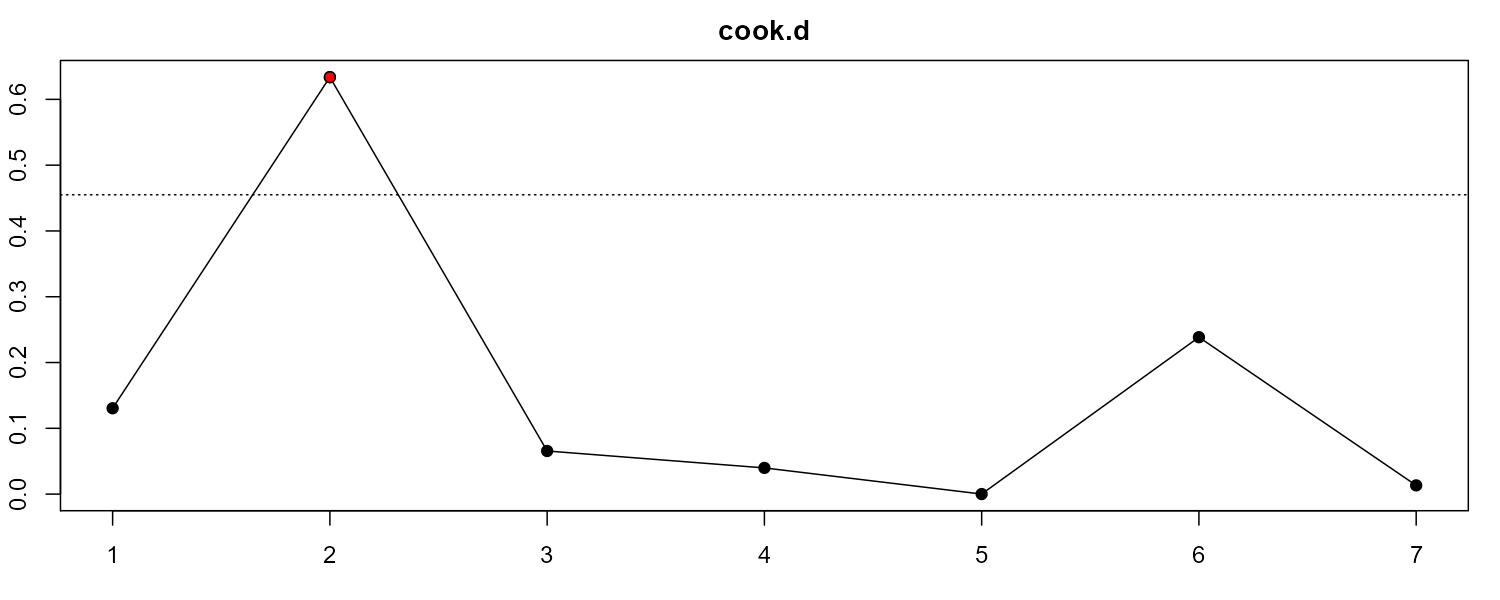


**Supplementary Figure 2.** *Cook’s distance values based on the random effects model of intrusion frequency. 1. Kleim et al., 2016 [1] 2. Porcheret et al., 2015 [2] 3. Porcheret et al., 2019 [3] 4. Wilhelm et al., 2021 [4] 5. Woud et al., 2018 Positive Cognitive Bias Modification [5] 6. Woud et al., 2018 Negative Cognitive Bias Modification [5] 7. Zeng et al., 2021 [6].*


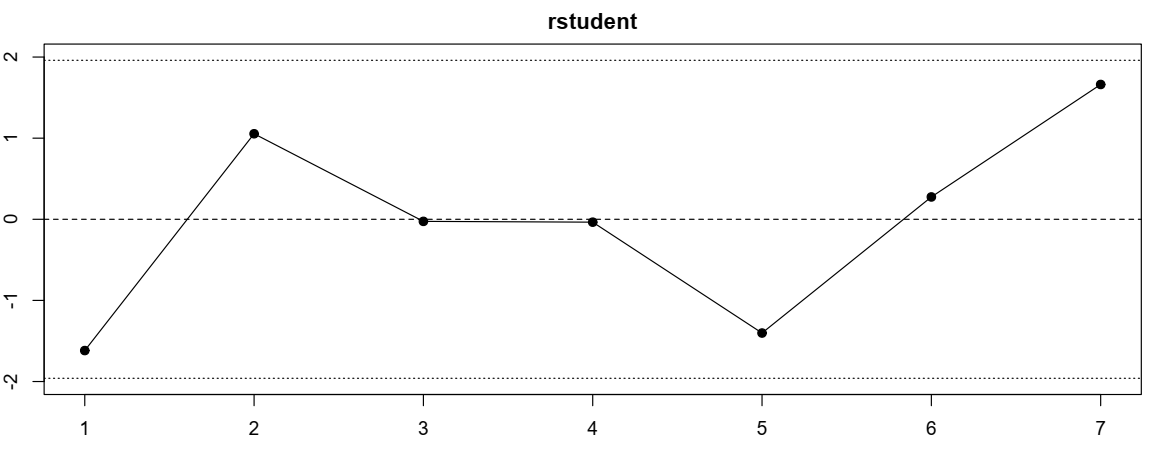
 **Supplementary Figure 3.** *Standardized residuals based on the random effects model of intrusion distress. 1. Kleim et al., 2016 [1] 2. Porcheret et al., 2015 [2] 3. Porcheret et al., 2019 [3] 4. Wilhelm et al., 2021 [4] 5. Woud et al., 2018 Positive Cognitive Bias Modification [5] 6. Woud et al., 2018 Negative Cognitive Bias Modification [5] 7. Zeng et al., 2021 [6].*


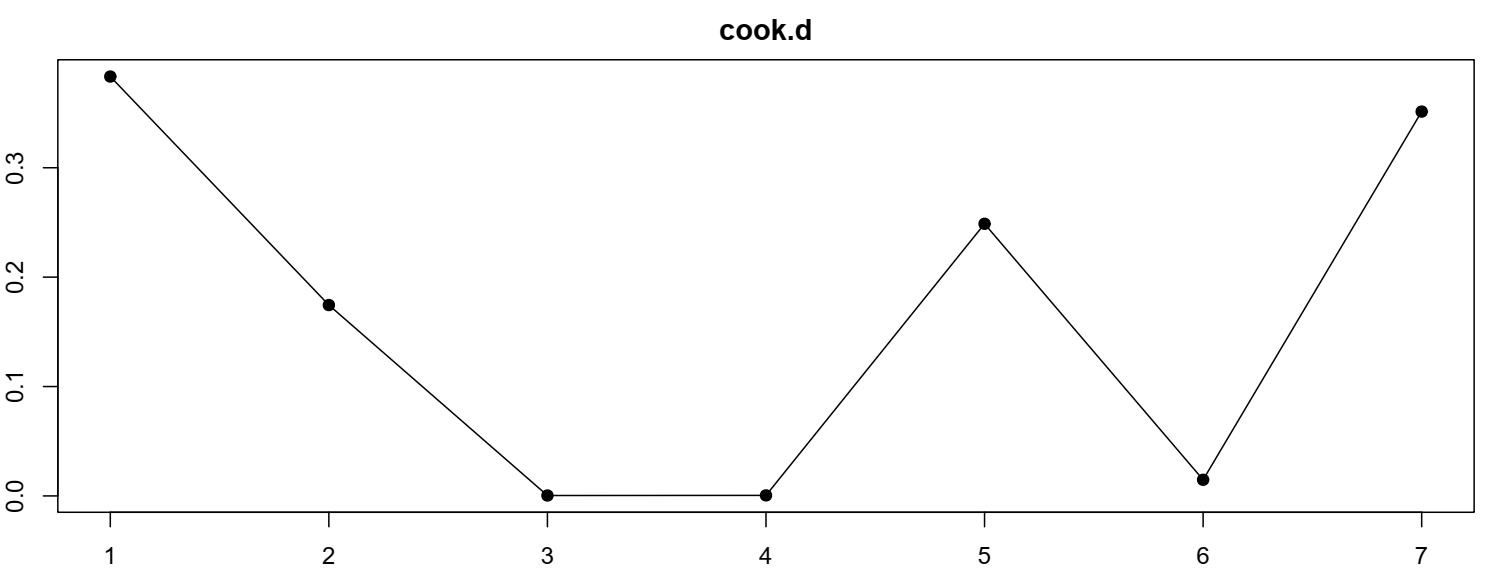


**Supplementary Figure 4.** *Cook’s distance values based on the random effects model of intrusion distress. 1. Kleim et al., 2016 [1] 2. Porcheret et al., 2015 [2] 3. Porcheret et al., 2019 [3] 4. Wilhelm et al., 2021 [4] 5. Woud et al., 2018 Positive Cognitive Bias Modification [5] 6. Woud et al., 2018 Negative Cognitive Bias Modification [5] 7. Zeng et al., 2021 [6].*

**References**

1. Kleim B, Wysokowsky J, Schmid N, Seifritz E, Rasch B. Effects of Sleep after Experimental Trauma on Intrusive Emotional Memories. *Sleep.* 2016;39(12):2125-2132. doi:[10.5665/sleep.6310](file:///C:\Users\psyc-pdn\Documents\Meta-analys%20Intrusions%20Sömn\10.5665\sleep.6310)

2. Porcheret K, Holmes EA, Goodwin GM, Foster RG, Wulff K. Psychological Effect of an Analogue Traumatic Event Reduced by Sleep Deprivation. *Sleep.* 2015;38(7):1017-1025. doi:[10.5665/sleep.4802](file:///C:\Users\psyc-pdn\Documents\Meta-analys%20Intrusions%20Sömn\10.5665\sleep.4802)

3. Porcheret K, van Heugten–van der Kloet D, Goodwin GM, Foster RG, Wulff K, Holmes EA. Investigation of the impact of total sleep deprivation at home on the number of intrusive memories to an analogue trauma. *Translational Psychiatry.* 2019;9(1):104. doi:[10.1038/s41398-019-0403-z](file:///C:\Users\psyc-pdn\Documents\Meta-analys%20Intrusions%20Sömn\10.1038\s41398-019-0403-z)

4. Wilhelm I, Azza Y, Brennwald K, Ehrt-Schäfer Y, Seifritz E, Kleim B. Investigating the effect of a nap following experimental trauma on analogue PTSD symptoms. *Scientific Reports.* 2021;11(1):4710. doi:[10.1038/s41598-021-83838-1](file:///C:\Users\psyc-pdn\Documents\Meta-analys%20Intrusions%20Sömn\10.1038\s41598-021-83838-1)

5. Woud ML, Cwik JC, Blackwell SE, et al. Does napping enhance the effects of Cognitive Bias Modification-Appraisal training? An experimental study. *PLOS ONE.* 2018;13(2):e0192837. doi:[10.1371/journal.pone.0192837](file:///C:\Users\psyc-pdn\Documents\Meta-analys%20Intrusions%20Sömn\10.1371\journal.pone.0192837)

6. Zeng S, Lau EYY, Li SX, Hu X. Sleep differentially impacts involuntary intrusions and voluntary recognitions of lab-analogue traumatic memories. *Journal of Sleep Research.* 2021;30(3):e13208. doi:[10.1111/jsr.13208](https://doi.org/10.1111/jsr.13208)

**Figure captions**

**Supplementary Figure 1.** *Standardized residuals based on the random effects model of intrusion frequency. 1. Kleim et al., 2016 [1] 2. Porcheret et al., 2015 [2] 3. Porcheret et al., 2019 [3] 4. Wilhelm et al., 2021 [4] 5. Woud et al., 2018 Positive Cognitive Bias Modification [5] 6. Woud et al., 2018 Negative Cognitive Bias Modification [5] 7. Zeng et al., 2021 [6].*

**Supplementary Figure 2.** *Cook’s distance values based on the random effects model of intrusion frequency. 1. Kleim et al., 2016 [1] 2. Porcheret et al., 2015 [2] 3. Porcheret et al., 2019 [3] 4. Wilhelm et al., 2021 [4] 5. Woud et al., 2018 Positive Cognitive Bias Modification [5] 6. Woud et al., 2018 Negative Cognitive Bias Modification [5] 7. Zeng et al., 2021 [6].*

**Supplementary Figure 3.** *Standardized residuals based on the random effects model of intrusion distress. 1. Kleim et al., 2016 [1] 2. Porcheret et al., 2015 [2] 3. Porcheret et al., 2019 [3] 4. Wilhelm et al., 2021 [4] 5. Woud et al., 2018 Positive Cognitive Bias Modification [5] 6. Woud et al., 2018 Negative Cognitive Bias Modification [5] 7. Zeng et al., 2021 [6].*

**Supplementary Figure 4.** *Cook’s distance values based on the random effects model of intrusion distress. 1. Kleim et al., 2016 [1] 2. Porcheret et al., 2015 [2] 3. Porcheret et al., 2019 [3] 4. Wilhelm et al., 2021 [4] 5. Woud et al., 2018 Positive Cognitive Bias Modification [5] 6. Woud et al., 2018 Negative Cognitive Bias Modification [5] 7. Zeng et al., 2021 [6].*
